# Complex Protein Retention Shifts with a Pressure Increase: An Indication of a Standard Partial Molar Volume Increase during Adsorption?

**DOI:** 10.1021/acs.analchem.2c01809

**Published:** 2022-09-20

**Authors:** Anja Kristl, Maja Caf, Matevž Pompe, Aleš Podgornik

**Affiliations:** † Institute of Forensic Medicine, Faculty of Medicine, University of Ljubljana, Korytkova ulica 2, Ljubljana 1000, Slovenia; ‡ Faculty for Chemistry and Chemical Technology, University of Ljubljana, Večna pot 113, Ljubljana 1000, Slovenia; § COBIK, Mirce 21, Ajdovščina 5270, Slovenia

## Abstract

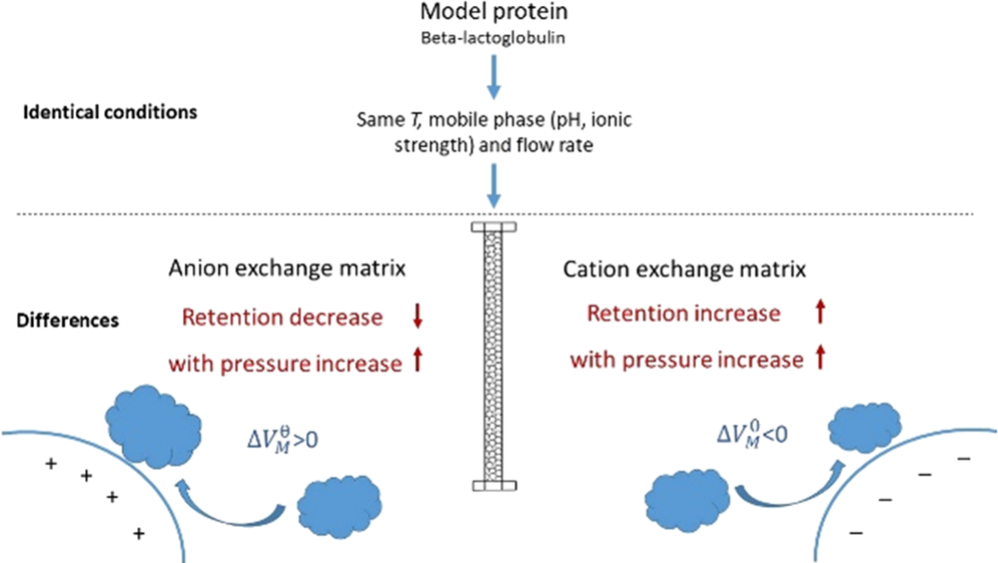

Studies of protein adsorption on reversed-phase and ion exchange stationary phases demonstrated an increase in retention with increasing pressure, which is interpreted as a standard partial molar volume decrease during the transition of the protein from a mobile to a stationary phase. Investigation of the pressure effect on the retention of lysozyme and IgG on a cation exchange column surprisingly revealed a negative retention trend with the increase of pressure. Further investigation of this phenomenon was performed with β-lactoglobulin, which enabled adsorption to be studied on both cation and anion exchange columns using the same mobile phase with a pH of 5.2. The same surface charge and standard partial molar volume in the mobile phase allowed us to examine only the effect of adsorption. Interestingly, a negative retention trend with a pressure increase occurred on an anion exchange column while a positive trend was present on a cation exchange column. This indicates that the interaction type governs the change in the standard partial molar volume during adsorption, which is independent of the applied pressure. Increasing the protein charge by decreasing the pH of the mobile phase to 4 reversed the retention trend (into a negative) with a pressure increase on the cation exchange column. A further decrease of the pH value resulted in an even more pronounced negative trend. This counterintuitive behavior indicates an increase in the standard partial molar volume during adsorption with the protein charge, possibly due to intermolecular repulsion of adsorbed protein molecules. While a detailed mechanism remains to be elucidated, presented results demonstrate the complexity of ion exchange interactions that can be investigated simply by changing the column pressure.

## Introduction

In recent years, the effect of pressure has become an important factor to consider in developing or adjusting separation conditions to meet desired criteria. As suggested by Giddings in 1966, an increase in pressure can improve the resolution between two solutes if their standard partial molar volume changes differently during the transition from a mobile to a stationary phase.^1^ Many researchers studying the reversed-phase (RP) separations later confirmed this effect when high-pressure UHPLC systems and columns were developed. The increase in pressure, always resulting in increased retention,^2−14^ was used to separate between small molecules with different sizes, shapes, and polarities.^2,7,11,15−18^ An even better distinction was possible when the retention behavior of macromolecules such as peptides and proteins was compared.^9,19−21^ When myoglobin was separated on an RP column, an increase in the retention time of up to 3000% was obtained when the pressure was increased to 1000 bar.^21^ An increase in retention of biopolymers and macromolecules with pressure was also demonstrated on an anion exchange column where up to an 80% increase in retention time and a 40% increase in resolution were observed. To understand these effects on RP or ion exchange (IEX) columns, the change in retention with pressure was described by equations based on basic thermodynamic principles^11,22−24^
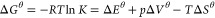
1where Δ*G*^θ^, Δ*E*^θ^, Δ*V*^θ^, and Δ*S*^θ^ are changes in the standard Gibbs free energy, internal energy, entropy, and volume of the system at a given temperature (*T*) and pressure (*p*). *R* is the gas constant, and *K* is the equilibrium constant. The effect of pressure on such a system can be studied by obtaining a partial derivative of eq 1 with respect to pressure at a constant temperature.^1^
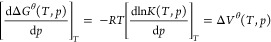
2

Equilibrium constant *K* can be expressed as the ratio between the distribution coefficient of the molecule and the displacing solvent *K*_M_/*K*_s_. The effect of pressure on solvent molecules is often considered negligible because of their small size, and *K*_s_ is therefore constant. Consequently, a change in *K*_M_ reflects the standard partial molar volume change Δ*V*_M_^θ^ of the molecule during the transition from the mobile to the stationary phase, representing a difference between the standard partial molar volume of the adsorbed molecule and its standard partial molar volume in the mobile phase. An increase in the distribution coefficient (*K*_M_) with pressure^2−14^ was therefore interpreted by a negative Δ*V*_M_^θ^, caused by a decrease of the standard partial molar volume during adsorption. Such a conclusion is also supported by high-pressure fluorescence measurements, which demonstrated a standard partial molar volume decrease during adsorption for protein staphylococcal nuclease.^25^

In the study of retention behavior at elevated pressure on an anion exchange column, it was shown that the pressure had very little to no effect on the interaction strength between the macromolecule and the stationary phase functional groups (parameter *A* in eq 3)—but for larger molecules, an increased number of binding sites was demonstrated.^22^ Since macromolecules such as proteins or peptides contain positive and negative charged moieties, pressure-induced conformational changes can expose additional charges on the macromolecule’s surface, potentially affecting interactions with a stationary phase. This structural change would therefore additionally contribute to the retention shift upon a pressure change, resulting in a non-linear trend according to eq 2. Such a behavior was indeed reported in few studies, including anion exchange interactions.^9,10^

In this work, we investigated the effect of pressure for cation exchange interactions. Initial investigations with IgG and lysozyme unexpectedly revealed a decrease in retention for both proteins upon a pressure increase. To further investigate this phenomenon, β-lactoglobulin (β-Lg) was used as a model protein. It is a globular protein with 162 amino acids, of which 18 residues can exhibit a positive charge and 25, a negative charge, depending on the pH, while the remaining are always neutral.^26^ More importantly, it has a high dipole moment^27^ and a broad isoelectric point between 4.8 and 5.9.^28^ This enables retention on an anion and cation exchange column at certain mobile phase pH values within this range. The same conditions in the mobile phase and the same pressure (same standard partial molar volume in the mobile phase and charge distribution) enabled the study of the change in the retention time trend with pressure solely due to the differences in the adsorption processes. To investigate the influence of the macromolecule’s charge on the observed pressure effect, we also performed experiments on the retention of β-Lg using mobile phases with pH values between 2 and 5.2, where β-Lg is in the form of a monomer at room temperature and a low concentration. Contrary, at higher pH values (7 and above), a dimeric form predominates.^28^

## Material and Methods

### Chemicals

Sodium chloride, sodium hydroxide, sodium phosphate monobasic of p.a. quality, and *o*-phosphoric acid (85%, HPLC) were purchased from Merck (Darmstadt, Germany). Hydrochloric acid and Tris–HCl of p.a. quality were purchased from Honeywell (Morris Plains, NJ, USA) and Kemika (Zagreb, Croatia), respectively. The deionized water was purified with a Milli-Q purification system from Millipore (Bedford, MA, USA) before use.

β-lactoglobulin of ≥90% purity, lysozyme from chicken egg white of ≥98% purity, and bovine serum IgG of ≥95% purity, all in the form of lyophilized powder, were purchased from Sigma-Aldrich.

### Preparation of Standard Solutions

β-lactoglobulin in a concentration of 1.8 mg/mL and both lysozyme and IgG in concentrations of 1 mg/mL were prepared by dissolving the lyophilized powder in mobile phase A. Before injection, solutions were left to stand refrigerated for 1 h and filtered through a 0.2 μm syringe filter.

### Instrumentation

The separations were performed using an Ultimate 3000 HPLC system (Thermo Fisher Scientific) equipped with a quaternary solvent delivery pump, an injector with a 100 μL loop, an autosampler with the temperature set to 10 °C, a column oven with a 6-port valve on each side, a diode array, and a conductivity detector connected to a pH meter. The experiments with β-lactoglobulin were performed on an analytical anion exchange column Proteomix SAX-NP3 with dimensions of 4.6 × 50 mm and 3 μm non-porous particles, and on a cation exchange column Agilent Bio SCX NP1.7 with dimensions of 4.6 × 50 mm and 1.7 μm non-porous particles. Columns can operate at pressures up to 10,000 psi (689 bar). The flow rate was set to 0.3 mL/min, and the column oven temperature was set to 25 °C. For lysozyme and IgG only, the cation exchange column was used. An increase of the column inlet pressure was achieved by fitting three restriction capillaries with a 25 μm ID (IDEX Health and Science, IL, US) and different lengths between two 6-port valves that were connected to the column outlet. Connection of restriction capillaries to the valves was established by coupling with Thermo Scientific SST Viper tubing (0.13 × 350 mm) and PEEK tubing of different lengths (0.125 mm ID). Tubes were connected by zero dead volume connectors. Regular PEEK tubbing (0.18 × 500 mm) was also used to connect the two valves without restriction tubbing, enabling separations at the fourth (regular) column inlet pressure. The tubing to the column inlet, after the column outlet, and between 6-port valves was kept in the column compartment to ensure a constant temperature. As described in previous work,^29^ a leak test was performed to ensure that the tubing connections were secure enough to withstand the pressure increase. All the connections passed the leak test.

Retention data were collected by Chromeleon 7.0 software (Thermo Fisher Scientific) and were corrected for the difference in the tubing length or so-called system transient time (extra-column volume). The column inlet pressures were obtained via the pump pressure sensor, located at the pump outlet and before the mixer, and calculated as described in the previous study.^22^

### HPLC Conditions

Isocratic separations of β-Lg were performed using 20 mM phosphate buffer at pH 5.2 as mobile phase/buffer A (MFA) and mobile phase/buffer B (MFB) that consisted of buffer A and 0.25 M NaCl at pH 5.2. The *v*/*v* % of buffer B was set at 25, 27, 29, 31, and 33 for experiments on both columns. Experiments at specific eluent compositions were repeated three times at four different column inlet pressures. Gradient runs were performed with same MFA but MFB with a higher NaCl concentration (MFA and 1 M NaCl). Gradient methods consisted of a linear gradient of a salt concentration, a wash (100% buffer B), and an equilibration step. Most gradient experiments were performed at pH 5.2 using different gradient slopes and two different (low–high) column inlet pressures on both columns. However, pH screening was performed only on the cation exchange column by using the same mobile phases A and B with lower pH values (2.0, 3.0, and 4.0).

Gradient runs of lysozyme and IgG were performed using 20 mM Tris–HCl buffer at pH 7.0 as mobile phase/buffer A and mobile phase/buffer B that consisted of buffer A and 1 M NaCl at pH 7.0. Both were eluted from the cation exchange column with a linear gradient slope of buffer B (3%/min) at two different (low-high) inlet pressures.

On account of higher sensitivity, the UV detector was set to measure absorbance at 220 nm. The mobile phase elution strength and pH value were monitored by a conductivity detector and a pH meter, respectively.

## Results and Discussion

**Figure 1:**
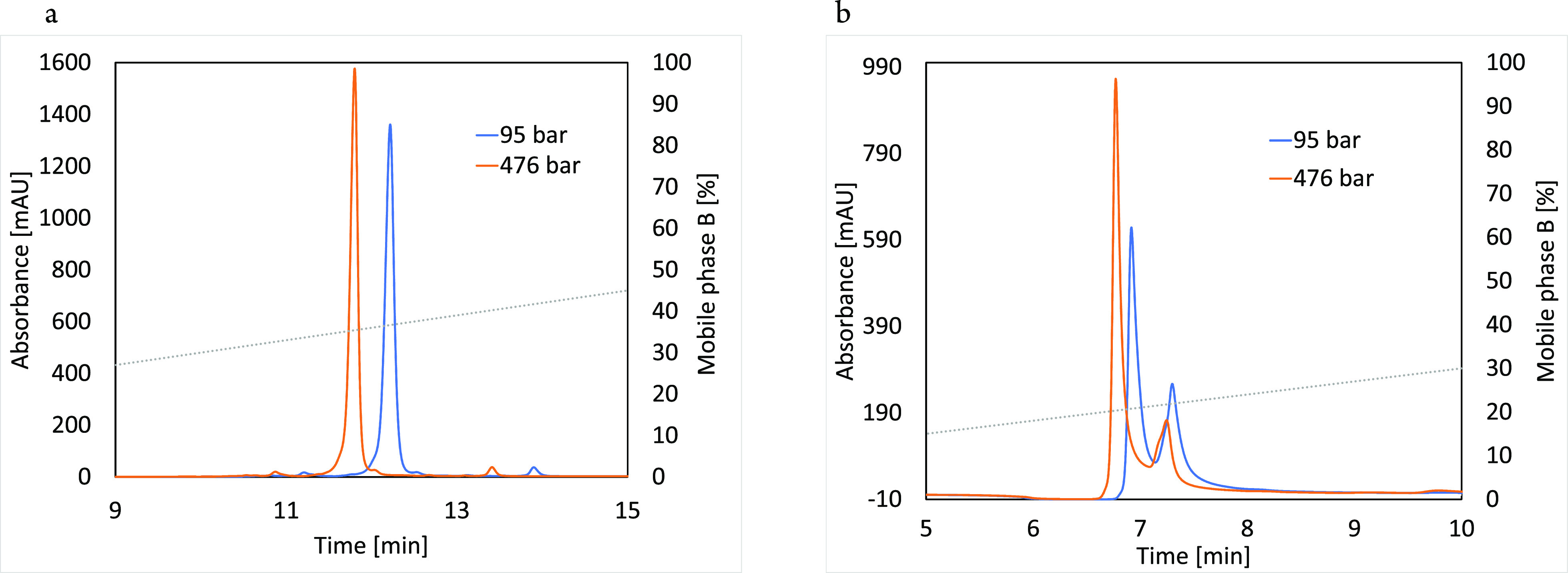
Gradient elution of lysozyme (a) and IgG (b) at low (blue) and high (orange) pressures on the CEX column. Mobile phase A was 20 mM Tris–HCl buffer, and B was A with 1 M NaCl, both at pH 7.0. Retention was investigated using a 3%/min gradient and the signal acquisition was set at 220 nm.

The effect of pressure on retention has already been studied extensively in RP and recently in anion exchange (AEX) chromatography. On the other hand, there are no reports on the pressure effect on cation exchange (CEX) interactions. To investigate whether these effects are different from those in AEX interactions, the retention of lysozyme was studied at two different pressures, 95 and 476 bar. Contrary to our expectations, a decrease in the retention time was observed (Figure 1a). This would indicate an unusual behavior, namely, an increase of the standard partial molar volume during adsorption, a phenomenon not reported so far. While no conformational changes are expected in solution at the applied pressure,^30^ lysozymes can lose their native structural stability when adsorbed onto well-defined homogeneous solid surfaces^31^ and they tend to undergo significant reorientation when adsorbed on a negative surface, thus affecting its desorption.^32^ This can lead to changes in the standard partial molar volume and consequently retention behavior during the pressure increase. If this is the cause of the observed decrease in retention with a pressure increase, such a trend might be a lysozyme peculiarity due to the structural changes when adsorbed and therefore would not be expected to occur for other macromolecules. To verify if this is the case, a similar experiment was performed with bovine IgG (Figure 1b).

Results demonstrated that a negative retention trend was also obtained for IgG, indicating that a negative retention trend can occur for various proteins and it might therefore be typical for CEX chromatography. To verify this hypothesis, one could evaluate adsorption behavior on CEX for many different proteins. However, this approach is limited to a fairly restricted group of proteins that have a high isoelectric point, which would enable their retention close to a neutral pH value on CEX. For other proteins, a low pH value would be required, potentially affecting their conformation and thus their biologic activity, limiting the validity of the obtained results.

A more elegant approach would be to investigate the retention of proteins that allow adsorption on CEX and also the AEX stationary phase under the same mobile phase conditions. This cannot be done with the tested lysozyme and IgG since retention on the AEX column could not be achieved under conditions where the proteins are retained on CEX resin. On the other hand, this behavior is expected for proteins that have a high dipole moment. There are not many such proteins available in quantities suitable for chromatographic studies, but fortunately, an example that has already been chromatographically well studied is β-lactoglobulin (β-Lg) isolated from cow’s milk.^33^ Due to its broad isoelectric point between 4.8 and 5.9^28^ and its high dipole moment (594 D),^27^ retention in this pH range has been reported to occur on both AEX and CEX stationary phases.^34^

**Figure 2:**
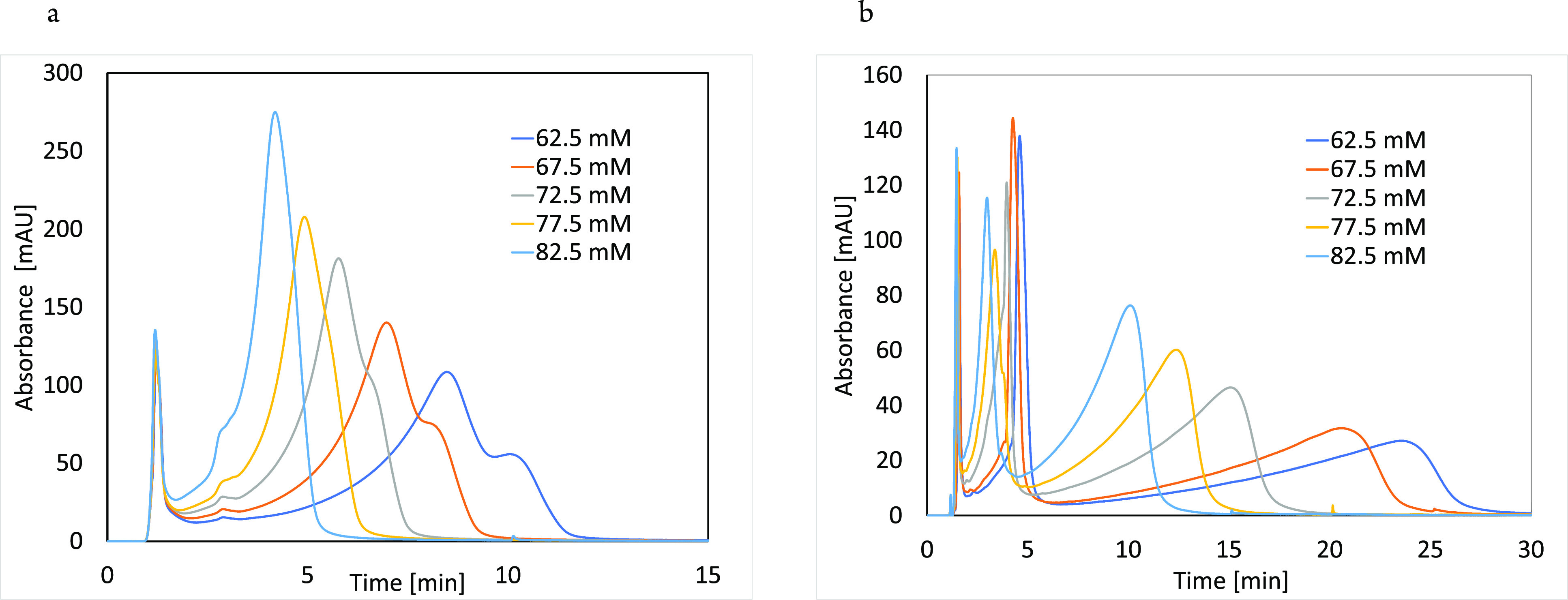
Isocratic experiments with β-Lg on CEX (a) and AEX (b) columns, varying the mobile phase NaCl concentration. The mobile phase consisted of 20 mM phosphate buffer at pH 5.2 and a NaCl concentration of 62.5, 67.5, 72.5, 77.5 and 82.5 mM. Signal acquisition was set at 220 nm.

To evaluate the pressure effect on β-Lg retention, the interaction with both CEX and AEX resin should be sufficiently strong. Therefore, several isocratic experiments were performed to estimate β-Lg retention without additional pressure increase. The mobile phase consisted of 20 mM phosphate buffer with a pH of 5.2 and different NaCl concentrations, namely, 62.5, 67.5, 72.5, 77.5, and 82.5 mM. Results are presented in Figure 2b.

Figure 2 shows that β-Lg splits into two peaks, indicating the separation of the two most abundant variants, namely, β-Lg A and β-Lg B. A better separation is seen on the AEX column, a result consistent with findings described by Yamamoto and Ishihara.^35^ β-Lg A has an aspartic acid residue at position 64 in the place of the glycine residue present in variant B. This additional negative charge is sufficient to enable separation on an AEX column and slightly decreases retention of β-Lg A on the CEX column. Since variant A is present in a much higher concentration in the sample solution, this chromatographic peak was evaluated in all further experiments.

To estimate the interaction of β-Lg with the stationary phase, retention factors *k* = (*t*_R_ – *t_m_*)/*t_m_* (*t*_R_ and *t_m_* are the retention and void time, respectively) were calculated and ln(*k*)–ln(*I*) plots were drawn (Figure S1), where *I* represents the elution concentration of NaCl. According to the stoichiometric displacement model (SDM),^36,37^ the average number of binding sites (*B*) and the interaction parameter (*A*) can be determined from the slope and intercept of eq 3
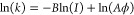
3where ϕ is the phase ratio (*V*_SF_/*V*_MF_). Results demonstrate that the β-Lg number of interaction sites on both columns is comparable: 3.18 on CEX and 3.83 on the AEX stationary phase. Based on the similar number of interaction sites, indicating similar surface coverage, it can be assumed that any effect of a pressure increase on retention can be compared on both columns.

**Figure 3:**
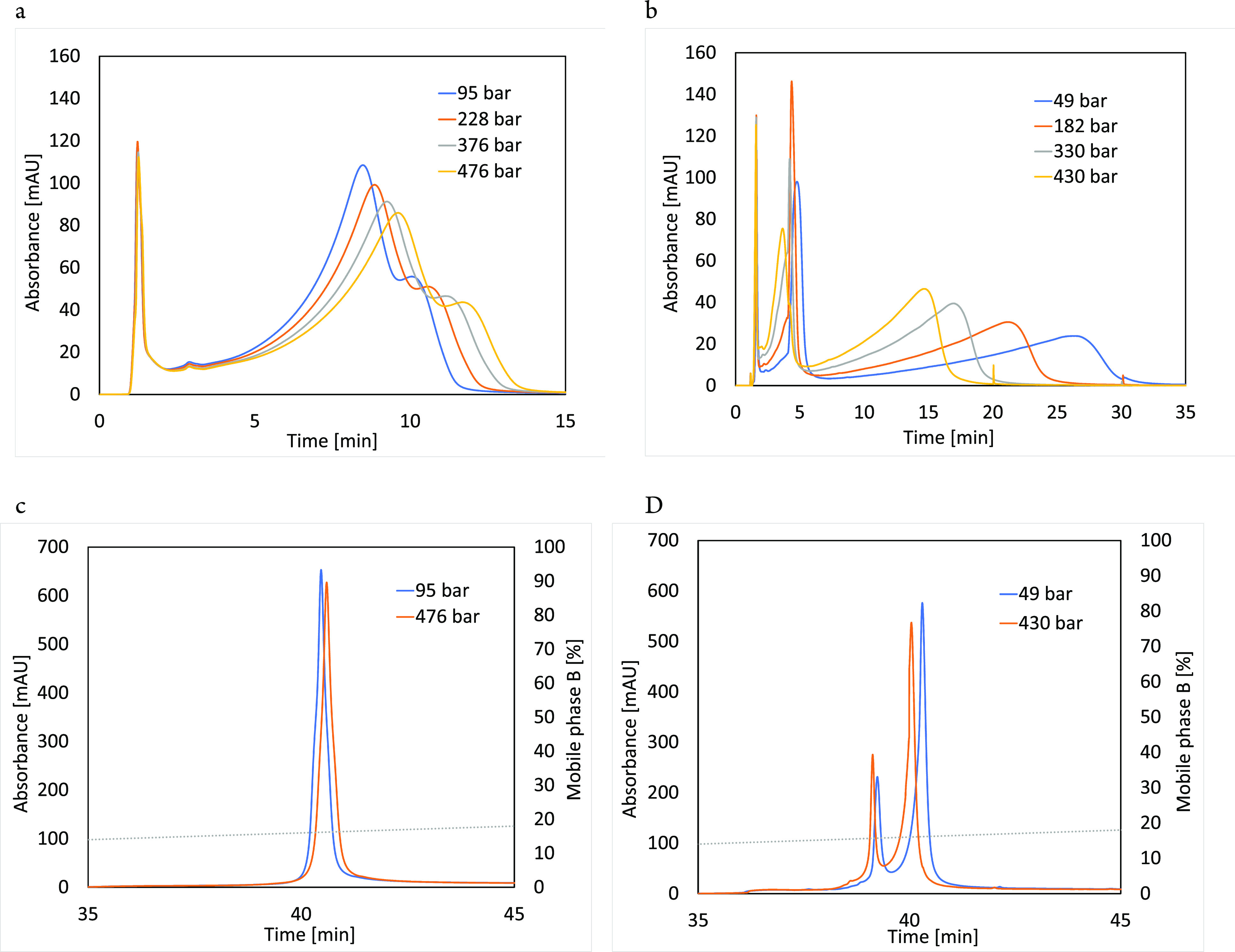
Isocratic experiments of β-Lg solution on CEX (a) and AEX (b) columns at four different column inlet pressures. The mobile phase consisted of 20 mM phosphate buffer at pH 5.2 and 62.5 mM NaCl for all experiments. Gradient experiments of β-Lg solution were also performed at low (blue) and high (orange) pressure on CEX (c) and AEX (d) columns with a 2.5%/min gradient. Mobile phase A was 20 mM phosphate, and B was mobile phase A with 1 M NaCl at pH 5.2. Signal acquisition was set at 220 nm.

The pressure effect was investigated by connecting the same restriction capillaries to the column outlets to increase the pressure on both columns equally. The CEX column was packed with smaller particles, resulting in a higher column inlet pressure of approximately 46 bar for all conditions. Since we focused on estimating the retention trend with a pressure increase, we did not adjust capillary length for the individual column pressure drop but maintained this difference between columns for all experiments. The comparison of isocratic β-Lg retention on AEX and CEX columns at the lowest mobile phase ionic strength (62.5 mM NaCl), which allows the strongest retention (Figure 2), is shown for different pressures in Figure 3.

It can be seen that pressure increase has a significant impact on the retention of β-Lg on both columns. On the CEX column (Figure 3a), we see a gradual increase in retention, the same trend as described in previous studies on IEX columns^22,29^ and by other researchers on RP columns,^9,23,24,38^ while opposite to the trend observed for lysozyme and IgG (Figure 1). At the highest pressure increase of 476 bar, the retention time of β-Lg on the CEX column increased by 1.14 min or 13.5% (0.93 or 14.4% increase in *k*). This demonstrated that retention on the CEX column does not necessarily cause a negative retention trend with a pressure increase. Interestingly however, the retention trend of β-Lg on the AEX column was negative, similar to the observed trend for lysozyme and IgG on CEX (Figure 1). Even at the lowest pressure increase of 182 bar, there was already a significant decrease in retention time by −5.21 min (−19.72% or −20.4% decrease in *k*), further decreasing by 9.41 min (−35.63% or −38.0% decrease in *k*) at 330 bar and even −11.75 min (−44.46% or −47.4% decrease in *k*) at 430 bar (Figure 3b**)**. This indicates that the negative retention trend with a pressure increase is not unique to CEX interactions but seems to be dependent on protein, mobile phase and IEX interaction type. The experiment was repeated for two pressures also in the gradient elution (Figure 3c,d), confirming trends from isocratic experiments.

As a decrease in the retention time with a pressure increase was not reported so far, we performed additional experiments to elucidate a possible mechanism of this phenomenon. First, we investigated whether the initial adsorption step is responsible for such trend or it develops during protein elution. As protein is strongly retained on the column during loading in gradient elution experiments, we were able to perform the loading under one pressure and the elution under a different pressure. Furthermore, the protein was left in the adsorbed state for 15 min, providing sufficient time for any surface reorientation, as has been reported for lysozyme.^32^ The same experiments were performed on both columns with each combination of high (476 or 430 bar) and low (95 or 49 bar) pressure: high *p* load to low *p* elute, high *p* load to high *p* elute, low *p* load to low *p* elute, and low *p* load to high *p* elute. The retention time of β-Lg under such retention conditions is given in Table 1.

**Table 1:** Retention Time of β-Lg on AEX and CEX Columns when Loading at Low (49 or 95 bar, Respectively)/High (430 or 476 bar, Respectively) Pressure and Eluting with Linear NaCl Gradient (2.5%/min, 20 mM Phosphate Buffer, pH 5.2), Again at High (430 or 476 bar)/Low (49 or 95 bar) Pressure

	β-Lg A retention time (min)	
	AEX column	CEX column
low *p* bind–low *p* elute	40.310	40.467
high *p* bind–low *p* elute	40.313	40.477
low *p* bind–high *p* elute	40.093	40.593
high *p* bind–high *p* elute	40.107	40.597

**Figure 4:**
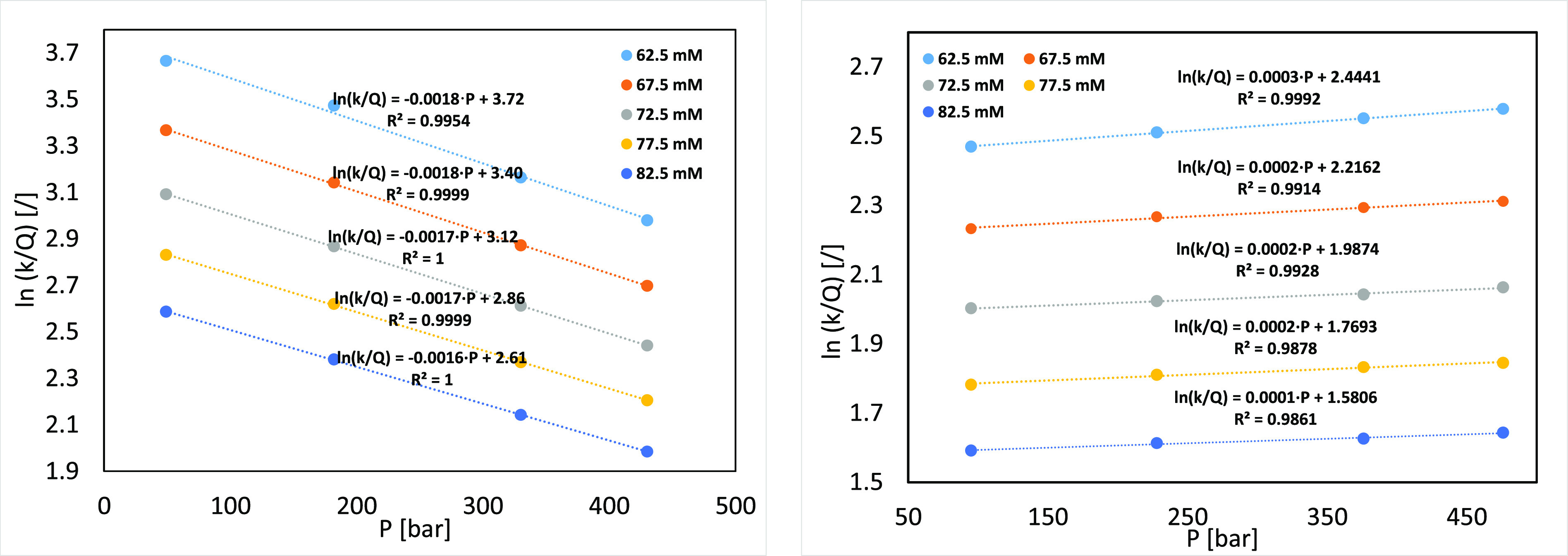
Logarithm of the distribution coefficient *K*_M_ (ln[*k*/ϕ]) as a function of pressure for AEX (left) and CEX (right) separations. The mobile phase consisted of 20 mM phosphate buffer at pH 5.2 and different NaCl concentrations. Signal acquisition was set at 220 nm.

It is evident that almost identical values were obtained at the same pressure during elution, regardless of the loading pressure. Furthermore, no reorientation changes in the adsorbed protein state were detected since the retention time change was the same as in the ordinary gradient elution experiments that are shown in Figure 3d, where there was no 15 min interval between the loading and elution. Therefore, only the pressure during migration affects the retention time, that is, when the protein is distributed between the two phases, which is consistent with the isocratic retention results. Based on consistency and reproducibility of the obtained results and no evidence of any irreversible changes during adsorption, it can be assumed that the decrease in β-Lg retention on the AEX column was caused by a pressure increase. Pressure change can affect retention in two ways: one is a linear change of the logarithm of the distribution coefficient *K*_M_, thermodynamically governed by a change in the standard partial molar volume during adsorption (eq 1), while another might be pressure-induced change in the protein conformation, exposing different groups on the protein surface and, with that, affecting its interaction with the stationary phase.^22^ This would result in a non-linear pressure dependence of the change in the logarithm of the distribution coefficient *K*_M_. In fact, a quadratic trend was observed in some experiments on RP^9,10^ and IEX columns,^22^ although in these cases, the retention increased with the pressure rise. To elucidate if this might be a possible explanation of the observed phenomenon, additional isocratic experiments were performed varying NaCl concentrations in the mobile phase at a pH of 5.2 to study the pressure effect on *K*_M_. The logarithm of the distribution coefficient *K*_M_ (ln[*k*/ϕ]) as a function of pressure for different mobile phase NaCl concentrations is presented in Figure 4.

Regardless of the mobile phase salt concentration and column type, the pressure dependence of ln*K*_M_ showed a linear trend (high *R*^2^) with a positive slope for CEX and a negative for AEX retention, as expected from the opposite trend in retention with pressure increase. Therefore, there is no indication of any conformational changes caused by elevated pressure, which is consistent with reports of β-Lg stability in this pressure range,^39^ most likely due to a rather small β-Lg molecular weight (18.4 kDa) and globular shape. Thus, the observed retention trend must be caused by the type of the interaction since the β-Lg standard partial molar volume in the solution is the same due to the identical mobile phase used for CEX and AEX experiments. The linearity (Figure 4) indicates that a standard partial molar volume change during adsorption is independent of the applied pressure and different retention trends on AEX and CEX columns demonstrate substantial differences in the adsorption mechanism, probably as a result of the different protein orientation on the surface.

Based on the obtained results it is reasonable to assume that the nature of the ion exchange interaction determines the changes in the standard partial molar volume during adsorption, resulting in a positive or negative retention trend with a pressure increase. If so, a change of the strength of the interaction should also have an impact on the retention behavior. This can be easily verified by changing the mobile phase pH value. While there should be no effect on the stationary phase charge since strong IEX groups were present on both columns, this is not the case for β-Lg. By examining the protein charge at different pH values determined from its amino acid structure, one can conclude that the interaction strength between the protein and stationary phase should be pH-dependent. Consequently, this might affect the standard partial molar volume change during adsorption and thus the retention trend with the pressure increase.

**Figure 5:**
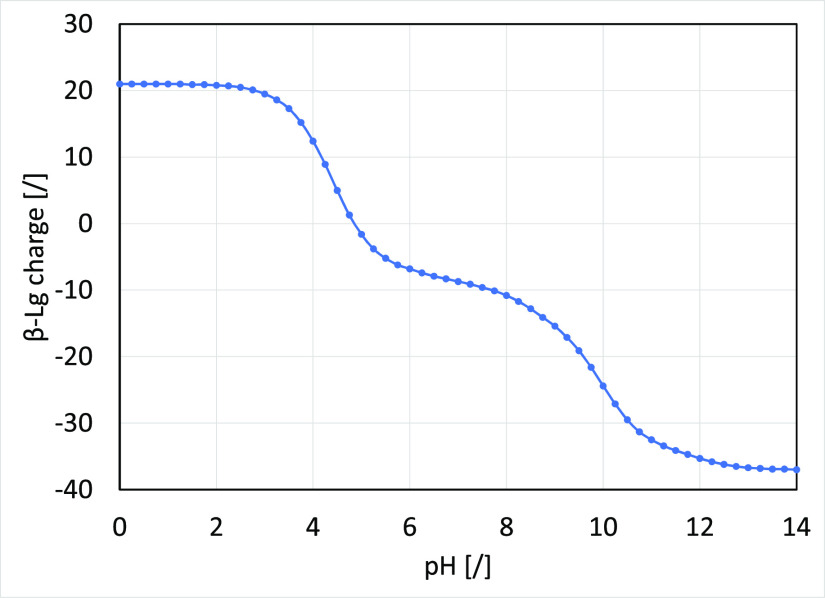
β-Lg A charge at different pH values. The curve was calculated from the β-Lg amino acid sequence and was calculated with a protein calculator.

Figure 5 shows that there is a moderate increase in the β-Lg negative charge with a pH value rise from 5.2 to 8.5. Unfortunately, contrary to lower pH values where the protein is present in a monomeric form, the dimeric form predominantly occurs in the discussed pH range,^28^ resulting in a significantly different size and also surface charge distribution. For this reason, any differences in the retention trend could not be attributed solely to a change of interaction strength, but structural changes must also be considered, making a correct interpretation challenging. Therefore, no further experiments were performed on the AEX column at higher pH values.

**Figure 6:**
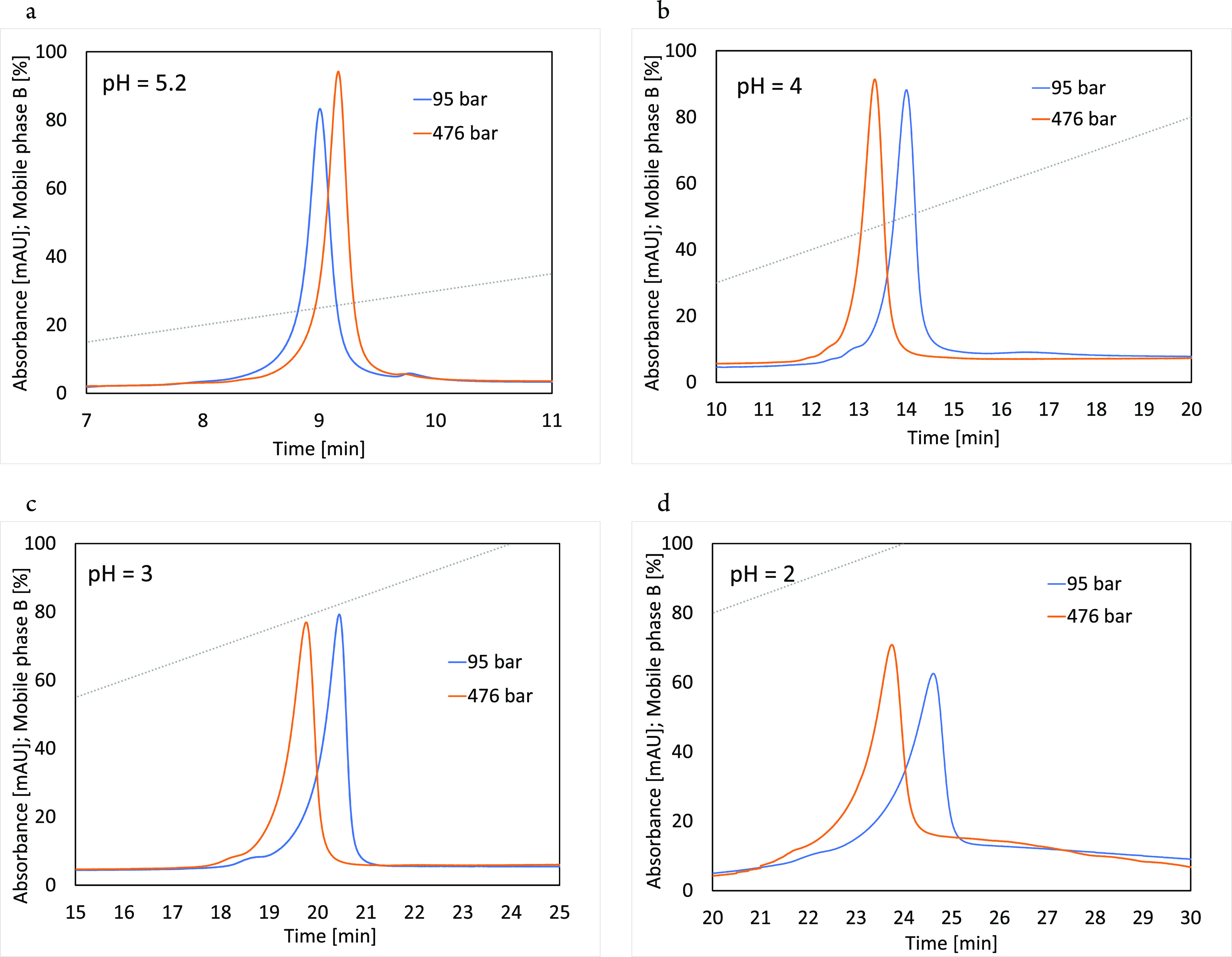
Gradient experiments of β-Lg solution at low (blue) and high (orange) pressure on the CEX column. Mobile phase A was 20 mM phosphate buffer, and B was A with 1 M NaCl at pH 5.2 (a), 4.0 (b), 3.0 (c), and 2.0 (d). Experiments were performed with a 5%/min gradient, and the signal acquisition was set at 220 nm.

On the other hand, at pH values between 2 and 5, β-Lg can form different aggregates, including dimers, tetramers, and even octamers, especially near the isoelectric point.^28^ However, in solutions with a low protein concentration at room temperature, it exists as a monomer,^28,40^ while its surface charge increases significantly with a decreasing pH. Therefore, a stronger interaction is expected, leading to a stronger retention on the CEX column.^33^ Chromatograms for different pH values are presented in Figure 6.

As expected, the change in the pH value has a significant impact on retention at normal pressure, which increases by lowering the mobile phase pH from 9.0 min at pH 5.2 to 14.0 min, 20.4 min, and 24.6 min for pH values 4, 3, and 2, respectively. More importantly, there is an additional influence on the retention trend with a pressure increase. While there was an expected positive retention shift of +0.158 min (+1.8%) at a pH of 5.2, a negative retention trend is observed already at a pH of 4.0, becoming even more pronounced with further lowering of pH, resulting in retention time decrease by −0.67 min (−4.76%), −0.68 min (−3.3%), and −0.87 min (−3.5%) at pH 4.0, 3.0, and 2.0, respectively. This indicates that indeed the interaction strength affects adsorption and the standard partial molar volume, but counterintuitively, in this particular case, a stronger interaction seems to reverse the retention trend and cause a higher specific protein volume in the adsorbed state. A possible explanation might be that a higher protein charge besides stronger interactions with the stationary phase also increases the repulsion between the adsorbed protein molecules, leading to a less dense packing on the surface and consequently higher standard partial molar volume. The effect of intermolecular repulsion was studied for a large protein catalase by varying the mobile phase ionic strength: directly by visualizing the surface coverage via liquid tapping-mode atomic force microscopy^41^ and indirectly for plasmid DNA by measuring the dynamic binding capacity.^42^ Both studies demonstrated that increasing the mobile phase ionic strength within a certain range causes shielding of the macromolecule’s charge, allowing tighter packing on the surface, for example, on the stationary phase. Of course, the mobile phase ionic strength also affects the interactions between the protein molecules in the mobile phase and also the organization of exchanged ions^43^ and thus the protein standard partial molar volume. Because of this dual effect, it is difficult to predict the outcome of the volume change; however, it should definitely be significant. Therefore, one would expect a difference in the change of the standard partial molar volume (between the free and adsorbed states) resulting in a different slope of the logarithm of the distribution coefficient versus pressure. Since the same mobile phase is used on both columns, the effect of ionic strength on the protein standard partial molar volume in solution is the same, so any changes in the slope should directly reflect differences in the standard partial molar volume in the adsorbed state. Indeed, a closer examination of the data in Figure 4 revealed differences in the slopes on the two columns. In both cases, the slope decreases with increasing the ionic strength from −0.0018 bar^–1^ (more precisely, −1.833165 × 10^–3^ bar^–1^) for 62.5 mM to −0.0016 bar^–1^ (more precisely, −1.584838 × 10^–3^ bar^–1^) for 82.5 mM (16% difference) for the AEX column and from 0.0003 bar^–1^ (more precisely, 2.85 × 10^–4^ bar^–1^) to 0.0001 bar^–1^ (more precisely, 1.29 × 10^–4^ bar^–1^), a 121% difference, for the CEX column. Since the experiments were performed at 25 °C, we can calculate the change of the standard partial molar volume (Δ*V*_M_) of β-Lg as presented in Table 2. Values from 45.4 to 39.3 cm^3^/mol on the AEX column and from −7.1 to −3.2 cm^3^/mol on the CEX column in 62.5 and 82.5 mM mobile phase were obtained, respectively. These values are of the same order of magnitude as values obtained for ion exchange interactions reported elsewhere but lower than values found in RP chromatography, where partial denaturation of proteins increases Δ*V*_M_ values.^21^ In all cases, however, a decrease of the standard partial molar volume during adsorption was reported.

**Table 2:** Changes in the Standard Partial Molar Volumes during Protein Adsorption Obtained for Different Interaction Types^a^

protein	interaction type	mobile phase	standard partial molar volume difference [cm^3^/mL]	reference
β-Lg	SAX	20 mM phosphate with 62.5 mM NaCl, pH 5.2	45.4	this work
β-Lg	SAX	20 mM phosphate with 67.5 mM NaCl, pH 5.2	43.7	this work
β-Lg	SAX	20 mM phosphate with 72.5 mM NaCl, pH 5.2	42.4	this work
β-Lg	SAX	20 mM phosphate with 77.5 mM NaCl, pH 5.2	40.8	this work
β-Lg	SAX	20 mM phosphate with 82.5 mM NaCl, pH 5.2	39.3	this work
β-Lg	SCX	20 mM phosphate with 62.5 mM NaCl, pH 5.2	–7.1	this work
β-Lg	SCX	20 mM phosphate with 67.5 mM NaCl, pH 5.2	–5.1	this work
β-Lg	SCX	20 mM phosphate with 72.5 mM NaCl, pH 5.2	–3.8	this work
β-Lg	SCX	20 mM phosphate with 77.5 mM NaCl, pH 5.2	–4.1	this work
β-Lg	SCX	20 mM phosphate with 82.5 mM NaCl, pH 5.2	–3.2	this work
thyroglobulin	SAX	20 mM Tris–HCl with 280 mM NaCl, pH 8.1	–36.0	(22)
BSA	SAX	20 mM Tris–HCl with 160 mM NaCl, pH 8.1	–32.1	(22)
lysozyme	RP	acetonitrile–trifluoroacetic acid	–110	(9)
lysozyme	RP	methanol–trifluoroacetic acid	–130	(9)
lysozyme	RP	acetonitrile–phosphoric acid	–97	(9)

a In studies where the change in the standard partial molar volume was pressure-dependent, values for the highest pressure are provided (SAX - strong anion exchange, SCX - strong cation exchange, and RP - reversed-phase).

It is interesting to evaluate what fraction of the standard partial molar volume of the protein represents the observed Δ*V*_M_ change during adsorption. Assuming that the change in the standard partial molar volume of β-Lg is independent of the applied pressure supported by a constant volume change at different pressures and considering the absolute value for β-Lg in solution (13505.6 cm^3^/mol),^44^ the standard partial molar volume increased by approximately 0.3% on the AEX column and decreased by approximately 0.04% on the CEX column during adsorption.

While further studies using techniques like high-pressure fluorescence spectroscopy^25^ are required to elucidate the exact mechanism causing this effect, the presented results demonstrated that positive or negative volume changes during adsorption can occur on the same column and protein when the pH is changed. This suggests that ion exchange chromatography is much more sensitive not only to the change in the standard partial molar volume but also to the specific changes at or near the adsorption site, such as charge, position, counter interactions, and similar. This pH dependence of the pressure effect on separations reveals the possibility of conditions that ideally compensate for the increase in retention time due to a standard partial molar volume change (typical compression upon adsorption) by a decrease in retention time due to a greater charge and less efficient packing on the surface making the transfer of the chromatographic method from HPLC to UPLC very robust. On the other hand, studies of the effect of pressure on retention provide a simple and very sensitive method to investigate small changes in the standard partial molar volume in the adsorbed state, even below 0.03%, and through that provide an insight into the adsorption mechanism.

## Conclusions

In this study, we demonstrated that the shift in protein retention due to an increase in pressure on an ion exchange column can occur in both directions. During isocratic experiments with β-Lg under same conditions on a cation and an anion exchange column, we observed a typical increase in the retention time on the cation exchange column but a decrease on the anion exchanger. However, a decrease of the mobile phase pH value reversed the trend from a positive to a negative one also on the cation exchange column. This demonstrates that a change in the pH can be used to tailor the retention shift with a pressure increase and indicates a possibility of a mobile phase pH value where the retention remains unaffected by the pressure. Such a method would be insensitive to pressure changes and could be transferred from an HPLC to a UHPLC system without modifications required to compensate for the effect of pressure change. On the other hand, adsorption studies performed under different pressures can serve as a simple and very sensitive method to study details of the adsorption mechanism of a particular macromolecule on various ion exchange columns under different mobile phase conditions.

## Supporting Information Available

Determination of β-Lg binding sites on CEX and AEX column (PDF)
