# Supplementary material for: Complex Protein Retention Shifts with a Pressure Increase: An Indication of a Standard Partial Molar Volume Increase during Adsorption?
Source: Anal Chem. 2022 Sep 20;94(39):13350–8. doi: 10.1021/acs.analchem.2c01809 (PMC9535627; doi:10.1021/acs.analchem.2c01809)
Supplement: Supplementary file 1 — ac2c01809_si_001.pdf [file ac2c01809_si_001.pdf]

Supporting Information

Complex protein retention shifts with pressure increase: an indication of standard partial molar volume increase during adsorption?

Anja Kristl[a,b], Maja Caf[b], Matevž Pompe[b], Aleš Podgornik[b,c]*

[a]Institute of Forensic Medicine, Faculty of Medicine, University of Ljubljana, Korytkova ulica 2, 1000 Ljubljana, Slovenia

[b]Faculty for Chemistry and Chemical Technology, University of Ljubljana, Večna pot 113, 1000 Ljubljana, Slovenia

[c]COBIK, Mirce 21, 5270 Ajdovščina, Slovenia

* correspondence: Aleš Podgornik, Faculty for Chemistry and Chemical Technology, University of Ljubljana, Večna pot 113, 1000 Ljubljana, Slovenia

Tel.: +386 1 479 8584

E-mail: ales.podgornik@fkkt.uni-lj.si

The file incudes raw data of β -Lg isocratic elution at different salt concentrations. The number of binding sites between the protein and the stationary phase on AEX and CEX column was obtained from the corresponding ln-ln plots of retention factor (*k*) versus salt concentration (*I*).

Table of content

- Figure S1: ln (*k*)-ln (*I*) plots determined from retention time of more abundant peak form Figure 2 S-2

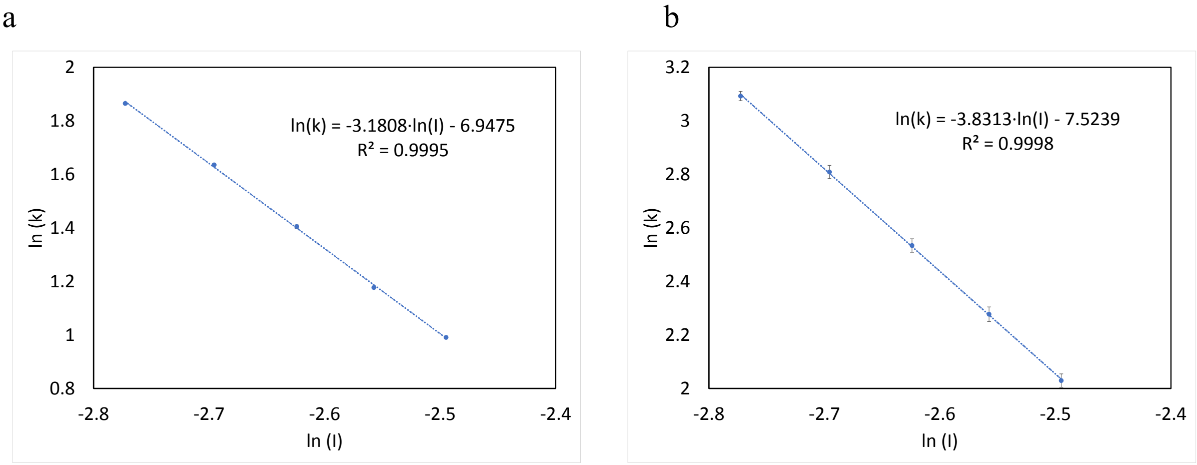

Figure S1: ln (*k*)-ln (*I*) plots determined from retention time of more abundant peak form Figure 2. Plot of values obtained from experiments on the (a) CEX and (b) AEX column (from Figure 2a and 2b, respectively).
